# Supplementary material for: Probability density and information entropy of machine learning derived intracranial pressure predictions
Source: PLoS One. 2024 Jul 1;19(7):e0306028. doi: 10.1371/journal.pone.0306028 (PMC11216561; doi:10.1371/journal.pone.0306028)
Supplement: Attachment — Submitted filename: ResponseToReviewers.pdf [file pone.0306028.s002.pdf]

RESPONSE TO REVIEWERS
PONE-D-23-31419: PROBABILITY DENSITY AND INFORMATION ENTROPY OF MACHINE LEARNING DERIVED INTRACRANIAL PRESSURE PREDICTIONS

ANMAR ABDUL-RAHMAN, WILLIAM MORGAN, ALEKSANDAR VUKMIROVIC, DAO-YI YU

We deeply appreciate the reviewers' generous contribution of time, valuable insights, and expert feedback. Their thoughtful critique significantly improved the communication of our findings to clinical researchers in the field. We believe the results of this paper provide a statistical method to validate the output of a machine learning-generated intracranial pressure prediction on an individual case basis, with potential applications in the management of patients with neurological and ophthalmic disorders. We implemented the reviewers' recommendations and hope that these changes meet the journal's requirements.

To facilitate the review, sentence line numbers are termed as **Revised Line...** to indicate the line number in the **manuscipt** document.

We have also color coded the typesetting in our response letter:

- The reviewer's comment is in red.
- The authors' response is in black.
- The changes in the manuscript in blue.

The references and equations cited in this response are numbered in sequence as they appear in the current document. The numerical sequence has been adjusted accordingly in the manuscript.

Response to Reviewer #1:

1. Page 4, ten lines from the bottom. "This is the case for intracranial pressure estimation, where a small margin of error would result in a significant difference in a clinical outcome."

This sentence seems rather important in the subsequent evaluation of model predictions, but there does not seem to be any motivation to support it. The Authors should expand this aspect of their presentation, possibly explaining if ICP is "more sensitive" to errors that other pressures in the eye (for instance, IOP), and, if so, why.

We are grateful for this insightful comment from reviewer #1. We wish to take the opportunity to expand on the recognised practical challenges in the precision and accuracy in ICP measurements. The following comment has been added to the introduction line 83 to 97:

There is a wide variability in normal intracranial pressure values largely due to differences in age, gender, and body mass index. Although normal values of 7-15mmHg have been reported. [1] In a prospective study of lumbar puncture measured ICP in 339 normal subjects, Bø et al. reported a reference range of approximately 3-22mmHg. [2]. Their findings suggest that physiological ICP may vary by up to a 7-fold range. Additionally studies demonstrate variation of continuously measured ICP within individuals, likely due to the multitude of interactions between physiologic parameters involved in intracranial pressure homeostasis, including postural, cardiovascular, neurological and respiratory parameters. [3] In a systematic review and meta-analysis of ICP monitoring systems Zaccharetti et al. found that the average error between simultaneous ICP measurements from different pressure sensors was approximately 1.5 mmHg, but the variability was large, with up to 11.4 mmHg difference in 95% of readings. [4] These factors render determining the precision and accuracy of ICP measurements challenging even in absence of underlying pathology.

2. Page 8, bottom. Comment on Figure 2.

Results seem to clearly indicate that the violin plots of the veins display a wider dynamic range than those of arteries, in both hold-out and cautionary subsets. It would be very interesting to see whether it may be possible to connect this behavior to the fluid-mechanical (rather different) characteristics of arteries and veins. This specific issue belongs to the more general question of how to combine physiology and machine-learning techniques, to devise an optimal methodology in the treatment and analysis of human data.

We appreciate reviewer #1's perceptive observation. We would like to discuss in further detail some reasons behind the differences in the dynamic range of pulsation values in the retinal vasculature. The following comment has been added to the discussion lines 511 to 539:

Differences in dynamic range in the pulsation parameters between the retinal veins and artery are highlighted both in Table 1 and Fig 2. These differences likely arise from structural and functional variability in the retinal vasculature and their characteristic interactions with pressurized anatomical chambers along their respective paths. The retinal arterioles lack an elastic lamina but possess a well-developed medial smooth muscle structure with 5-7 layers. In contrast, retinal veins have thinner walls (13.929 ± 0.041 μm) compared to arteries (17.559 ± 0.062 μm), and a thinner muscle layer of 3-4 layers that transitions to fibroblasts near the optic disc [5]. These structural differences influence the compliance and impedance characteristics of the retinal vasculature. While direct measurements of compliance and incremental modulus of elasticity (E_{inc}) in the retinal vasculature are lacking, inferences from systemic vessels can be drawn, where veins exhibit higher compliance, with a sigmoidal pressure-volume curve compared to the curvilinear relationships seen in arteries [6–11]. These differences in compliance likely lead to differences in pulsation characteristics. Although physiological interactions between the retinal vasculature with

pressurized anatomical chambers: intracranial, intraorbital, and intraocular spaces are yet to be fully understood. Our recent work highlights previously unrecognized and clinically significant interactions of the retinal arterioles with intracranial pressure, evidenced by the ability to generate intracranial pressure predictions from the arterial tree with an accuracy comparable to that of the retinal veins [12]. However, phase relationships with intracranial pressure remain unknown in this part of the retinal vascular system. In contrast, the intracranial pressure wave may dominate the pulse frequencies in the structurally thinner and higher compliance retinal venous wall, potentially explaining the higher venous Fourier coefficients, higher HRW_a, and its attenuation characteristics [13,14]. Observed differences in the phase of the retinal vascular pulse support this explanation, with experimental data showing phase congruence between retinal venous pulsation and intracranial pressure. [15] Future research in this field is recommended to shed light on the physiological interactions of the retinal vessels with anatomically related pressurized chambers.

3. Page 9, Table 1.

It is interesting to hear the Authors' opinion about whether the knowledge of more than two Fourier coefficients in the HWA may provide a better accuracy of the descriptive statistics. If so, as it appears to be reasonable, how difficult might be the extension of the present approach to the evaluation of the first N Fourier coefficients, N being the first integer such that the coefficient a_{N+1} b_{N+1} is smaller than a given tolerance.

Reviewer #1 raises a fascinating question regarding the limit of harmonics that can be detected from the retinal circulation. Although theoretically there are an infinite number of Fourier harmonics that can be extracted from a signal, practically this is limited by both the finite signal duration and the signal-to-noise ratio. For a practical signal, only a finite window in time can be considered, limiting the highest frequency that can be accurately

represented using Fourier analysis. This relationship is encapsulated by the Heisenberg-Gabor uncertainty principle [16]. The principle asserts that there is a tradeoff between temporal (Δt) and spectral (Δf) resolution, which is represented by the mathematical identity $\Delta f \cdot \Delta t \geq C$, where C is a constant. [17] Accordingly, the shorter the time window, the higher the detectable frequencies, but this comes at a cost to accuracy. Moreover, as the signal-to-noise ratio decreases (more noise), the higher frequency harmonics become increasingly unreliable due to noise contamination. At some point, the higher frequencies become indistinguishable from pure noise and convey no meaningful information about the original signal. Therefore, in practice, the effective limit of the number of harmonics that can be considered is based on signal duration and the signal-to-noise ratio to ensure reliable results.

By extension from what is known about the harmonics of the systemic circulation, which have been studied more thoroughly compared to the ophthalmic circulation, it has been reported that the first two harmonics account for approximately 85% of the pulsatile portions of pressure and flow waves in large arteries [18]. Krovetz et al. analyzed the harmonics of intracardiac and arterial pressure waves, reporting that 3-10 harmonics were required to reproduce measured pressure waveforms. However, theoretically derived differential pressures and derivatives appeared to contain six-fold the number of higher-frequency harmonics [19]. Despite this theoretical estimate, there is a fundamental limit to the number of harmonics extracted from measured pressure-flow waves. Beyond the tenth harmonic component, the harmonic magnitudes of the aortic pressure pulse and aortic flow pulse are negligible due to sampling factors and signal noise. A further limit is imposed by the Nyquist criterion, which requires a sampling frequency of at least twice the highest frequency content to accurately reconstruct the original waveform. Due to a low signal-to-noise ratio, Milnor demonstrated that harmonics above 25 Hz are unreliable. Practically, the noise level and

resolution of recording instruments reduce the accuracy of harmonics beyond the sixth, and measurements are, therefore, subject to uncertainty at higher harmonics [20–23]. There are other considerations specific to the retinal circulation; the vessel wall and fluid viscosities attenuate the pulse wave with centrifugal propagation. The attenuation, or damping, is more significant at higher frequencies, a phenomenon known as dispersion. For example, the incisura is progressively damped as it propagates. Indeed, when the pulse reaches the femoral artery, the characteristic high-frequency features of the aortic pulse disappear, and instead, a smooth waveform is seen when the pulse reaches the arterioles. Damping results in an approximately sinusoidal waveform; therefore, higher harmonics may become increasingly negligible peripherally. Specific to ophthalmic imaging, optical and motion artifacts coupled with the low amplitude vascular pulse impose further limits on the Fourier harmonics that can be effectively extracted from the retinal circulation [24].

MINOR ISSUES:. 1. As a general comment, it appears that the acronym are rarely defined on their first occurrence, rather, their definition is made in the caption of the tables and/or figures. This approach seems to confer a touch of "confusion" to the presentation and, likely, could be amended by defining properly each quantity when it is mentioned for the very first time.

We apologize for this oversight. The abbreviations have been reviewed and clarifications have been applied accordingly particularly in the materials and methods section.

2. Page 3, line 12 from top: "it is dependent on the" —-> "is dependent on the"

4 lines from bottom: "weights should, by definition, should coincide." —-> "weights should, by definition, coincide."

Corrected line 42.

Corrected line 57.

3. Page 4: ”between a continuous random variable (X)”: the letter ”X” should be non-capitalized.

”The function (f(x)) is piecewise continuous.”: why ”(f(x))” and not ”f(x)”??

Corrected line 71.

Corrected line 72 and 75.

4. Page 6: ”A total of A total of 129,600 data points” —-> ”A total of 129,600 data points”

”conflicting predictions from the arterial and venous models and were found in ongoing evaluation of the model.” —-> ”conflicting predictions from the arterial and venous models were found in ongoing evaluation of the model.”

Corrected line 161.

Corrected line 169.

5. Page 10, line 1: ”of the hold-out test cases. Whereas the median” —-> ”of the hold-out test cases, whereas the median”

The punctuation has been corrected line 256.

6. Page 18, ten lines from bottom: ”The analysis detailed in chapter ??” this unresolved reference must be fixed.

The reference has been corrected line 470.

Response to Reviewer #2:

Reviewer #2: Recommendation is to include to a list of references and to study at least the following papers: S.T. van Hal et al . Neurocrit Care. https://doi.org/10.1007/s12028-023-01910-2 ; Jue Wang et al. BMC Medical Informatics and Decision Making. https://doi.org/10.1186/s12911-023-02247-8;

We would like to thank reviewer #2 for drawing our attention to recent publications in the field we have added the following comment in the introduction lines 97 to 114:

While evidence suggests artificial intelligence (AI) algorithms could aid clinical decision-making, current research emphasizes the need for systems enabling model evaluation, bias detection, and generalizability. Wang et al. compared machine learning models to traditional scoring tools for predicting mortality risk in traumatic brain injury patients. Among 47 studies with 156 models, machine learning models showed relatively high accuracy for both in-hospital and out-of-hospital mortality. Notably, traditional tools achieved comparable accuracy. The authors highlight the need for standardized reporting and validation of machine learning models to ensure clinical applicability and generalizability. [25] Similarly, van Hal et al. assessed bias risk and clinical readiness of studies using AI to predict intracranial hypertension in traumatic brain injury patients. They found most studies had high bias risk and low readiness for clinical integration. Despite promising potential, the authors concluded further improvement and validation are necessary before implementing these models in clinical practice. [26] These findings underline the practical importance of bias detection, as AI algorithms may perform well under specific conditions not replicable in clinical settings. Using properties of the probability density function the approximation of the estimate to the ground truth may be confirmed by the capacity to validate ICP readings, irrespective of the measurement methodology.

1. Other Corrections

(1) Line 147 mentions written consent was obtained from participants.

(2) Line 157 ethics committee project number added.

(3) Line 200 the variable x in the equation changed to lowercase.

(4) Line 376 "Whereas in the venous model..." changed to "While in the venous model..."

References

1. Munakomi S, Das M. Intracranial Pressure Monitoring. StatPearls [Internet]. 2019;.
2. Bø SH, Lundqvist C. Cerebrospinal fluid opening pressure in clinical practice–a prospective study. Journal of Neurology. 2020;267:3696–3701.
3. Jonas JB, Wang N, Yang D, Ritch R, Panda-Jonas S. Facts and myths of cerebrospinal fluid pressure for the physiology of the eye. Prog Retin Eye Res. 2015;46:67–83.
4. Zacchetti L, Magnoni S, Di Corte F, Zanier ER, Stocchetti N. Accuracy of intracranial pressure monitoring: systematic review and meta-analysis. Crit care. 2015;19:1–8.
5. Hogan MJ, Feeney L. The ultrastructure of the retinal blood vessels: I. The large vessels. J Ultrastruct Res. 1963;9(1-2):10–28. doi:https://doi.org/10.1016/s0022-5320(63)80033-7.
6. Nichols WW, Edwards DG. Arterial elastance and wave reflection augmentation of systolic blood pressure: deleterious effects and implications for therapy. J Cardiovasc Pharmacol Ther. 2001;6(1):5–21. doi:https://doi.org/10.1177/107424840100600102.
7. Moreno AH, Katz AI, Gold LD, Reddy R. Mechanics of distension of dog veins and other very thin-walled tubular structures. Circ Res. 1970;27(6):1069–1080. doi:https://doi.org/10.1161/01.RES.27.6.1069.
8. Keener J, Sneyd J. The circulatory system. In: Mathematical Physiology II: Systems Physiology. New York: Springer-Verlag; 2009. p. 471–522.
9. Feher JJ. Vascular function: Hemodynamics. In: Feher JJ, editor. Quantitative Human Physiology: An introduction. Cambridge, MA, United States: Elsevier-Academic Press; 2012. p. 498–507.
10. Burton AC. Arrangements of Many Vessels. In: Physiology and biophysics of the circulation: an introductory text. 2nd ed. Chicago, United States: Year Book Medical Publishers; 1972. p. 51–62.
11. Caro C, Pedley T, Schroter R. The systemic veins. In: The mechanics of the circulation. Cambridge, United States: Cambridge University Press; 2012. p. 426–466.
12. Abdul-Rahman A, Morgan W, Yu DY. A machine learning approach in the non-invasive prediction of intracranial pressure using Modified Photoplethysmography. PLoS One. 2022;17(9):e0275417.
13. Abdul-Rahman A, Morgan W, Yu DY. Measurement of normal retinal vascular pulse wave attenuation using modified photoplethysmography. PLoS One. 2020;15(5):e0232523.

14. Abdul-Rahman A, Morgan W, Jo Khoo Y, Lind C, Kermode A, Carroll W, et al. Linear interactions between intraocular, intracranial pressure, and retinal vascular pulse amplitude in the Fourier domain. PLoS One. 2022;17(6):e0270557.
15. Morgan WH, Lind CR, Kain S, Fatehee N, Bala A, Yu DY. Retinal Vein Pulsation Is in Phase with Intracranial Pressure and Not Intraocular Pressure. Invest Ophthalmol Vis Sci. 2012;53(8):4676–4681. doi:https://doi.org/10.1167/iovs.12-9837.
16. Folland GB, Sitaram A. The uncertainty principle: a mathematical survey. J Fourier Anal Appl. 1997;3(3):207–238.
17. Hsieh IH, Saberi K. Imperfect pitch: Gabor's uncertainty principle and the pitch of extremely brief sounds. Psychon Bull Rev. 2016;23(1):163–171.
18. Wiener F, Morkin E, Skalak R, Fishman AP. Wave propagation in the pulmonary circulation. Circ Res. 1966;19(4):834–850.
19. Krovetz LJ, Goldbloom SD. Frequency content of intravascular and intracardiac pressures and their time derivatives. IEEE. 1974;(6):498–501.
20. Li J. Physical concepts and basic fluid mechanics. In: Dynamics of the vascular system. vol. 1. Singapore: World Scientific Publishing Co Pte Ltd; 2004. p. 41–75.
21. Qureshi MU, Colebank MJ, Schreier DA, Tabima DM, Haider MA, Chesler NC, et al. Characteristic impedance: frequency or time domain approach? Physiol Meas. 2018;39(1):014004.
22. Milnor WR. Hemodynamics. Cardiac dynamics. 1989;.
23. Li JK. Arterial Pulse Transmission Characteristics. In: The Arterial Circulation: Physical Principles and Clinical Applications. Springer Science & Business Media; 2000. p. 69–128.
24. Abdul-Rahman A. Retinal Vascular Pulse Wave Analysis in the Fourier Domain. PhD Thesis. 2023;.
25. Wang J, Yin MJ, Wen HC. Prediction performance of the machine learning model in predicting mortality risk in patients with traumatic brain injuries: a systematic review and meta-analysis. Bmc Med Inform Decis. 2023;23(1):142.
26. van Hal S, van der Jagt M, van Genderen M, Gommers D, Veenland J. Using Artificial Intelligence to Predict Intracranial Hypertension in Patients After Traumatic Brain Injury: A Systematic Review. Neurocrit Care. 2024; p. 1–12.
